# Cell manufacturing for cell-based tissue engineering: a focus on vascularized, skeletal muscle regeneration

**DOI:** 10.3389/fceng.2025.1637075

**Published:** 2025-08-06

**Authors:** Bruno de Medeiros Cartaxo Esmeraldo, Keely A. Laurence, Brian J. Kwee

**Affiliations:** Department of Biomedical Engineering, University of Delaware, Newark, DE, United States

**Keywords:** cellular heterogeneity, potency, cell identity, cell manufacturing, tissue engineering, vascularization, muscle regeneration

## Abstract

Cell manufacturing processes play a crucial role in cell-based tissue engineering by isolating, purifying, culturing, expanding, modifying, cryopreserving, and formulating patient-derived cells *in vitro* before utilizing them for tissue regeneration. Currently, researchers apply various methods for cell manufacturing, including bioreactors, defined chemical cues, and substrate modifications. However, factors such as loss of cell potency and heterogeneity are critical challenges when engineering tissues for regenerative medicine. In particular, neglecting cellular heterogeneity during cell expansion prevents the formation of tissues that recapitulate the structural and cellular heterogeneity of our native tissues. This review discusses current and emerging approaches for cell manufacturing, with a focus on biomanufacturing for vascularized, skeletal muscle tissue engineering. Specifically, this review highlights 1) the U.S. Food and Drug Administration’s regulation of manufacturing for cell therapies, 2) state-of-the-art approaches for manufacturing endothelial cells and muscle stem cells that maintain cellular identity and potency, and 3) emerging tools and methods for measuring and manipulating cellular heterogeneities. Ultimately, these approaches can be leveraged to manufacture and formulate tissue-engineered products that mimic the heterogeneous form and function of our native tissues.

## Introduction

Cell-based tissue engineering broadly encompasses approaches where patient-derived cells regenerate tissues and organs with or without biomaterial scaffolds. Biomaterial scaffolds, which may consist of synthetic engineered materials or naturally derived extracellular matrix proteins, can provide microenvironments for the cells to proliferate, differentiate, and self-assemble into *de novo* tissues. These general approaches have demonstrated some success in regenerating tissues that are functionally similar to their *in vivo* counterparts in preclinical models. For example, several groups have successfully demonstrated the ability to engineer vascularized skeletal muscle tissue from endothelial cells and muscle stem cells to treat volumetric muscle loss (Levenberg et al., 2005; Quarta et al., 2017; Nakayama et al., 2019). However, the clinical success of these cell-based tissue engineering approaches has been limited. Current U.S. FDA-approved, cell-based tissue-engineered products for regenerative medicine mainly consist of avascular tissues with relatively simple form and function (Table 1).

A critical barrier to the success of these cell-based tissue engineering approaches is the manufacturing of the regenerative cells. Cell manufacturing broadly includes the processes of cell isolation, purification, culture, expansion, modification, cryopreservation, and formulation of the cell-containing product (i.e., combination with a biomaterial). A widely recognized challenge in manufacturing cells for regenerative medicine and tissue engineering involves exponentially expanding the cells while maintaining their identity and potency. Cell identity can generally be defined by the cell type and the cell state, which includes the form and function of the cell (Mincarelli et al., 2018). Moreover, cellular potency is generally defined as the ability of a specific cell product, when used in a therapeutic context such as bolus delivery or within a device, to perform a desired therapeutic effect (Singh et al., 2016). During cell culture and expansion, cell identity and potency are often altered or lost during *in vitro* culture conditions that do not recapitulate the cells’ native *in vivo* settings (Gilbert et al., 2010; Richter et al., 2021).

A unique cell manufacturing challenge for engineering tissues that model their native *in vivo* counterparts is the need to measure and maintain cell heterogeneity. Our native tissues and organs are highly heterogeneous in their structure and function, which can be attributed to single-cell level heterogeneities. For example, primary endothelial cells derived from vascular tissues and muscle stem cells derived from skeletal muscle are recognized to be highly heterogeneous on a single-cell level (Cho and Doles, 2017; Barruet et al., 2020; Li et al., 2021). Endothelial cell heterogeneity can be attributed to the different types of blood vessels the cells are derived from, which include vessels with varying geometry and function (i.e., capillaries, arterioles, venules, arteries, and veins) (Dumas et al., 2021; Gunawardana et al., 2021). Furthermore, muscle satellite cells are heterogeneous in terms of their differentiation potency and whether they are derived from fast or slow-twitch muscles (Petrany et al., 2020). Various muscle fiber structures support diverse muscle functions, such as differences between the soleus muscle (which sustains posture, predominantly slow-twitch fibers) and the gastrocnemius muscle (which facilitates movement, similar composition of slow and fast-twitch fibers) (Schiaffino and Reggiani, 2011; Schoenfeld et al., 2020).

During conventional cell manufacturing processes, single-cell heterogeneities are frequently overlooked, and the cells are treated as a homogenous population. Ignoring these heterogeneities during cell manufacturing processes often leads to homogenous, engineered tissues that do not model the functional and structural heterogeneities of native tissues. For example, self-assembled vasculature in biomaterials is often disorganized, due to the stochastic assembly of blood vessel networks of varying blood vessel length, diameter, branching index, and orientation (Morgan et al., 2019). Furthermore, self-assembled muscle fibers in biomaterials often yield a random distribution of myofibers with varying length and diameter (Madden et al., 2015; Rao et al., 2018). Thus, there is a critical need for novel approaches that can measure, maintain, and manipulate cellular heterogeneities in cell-based tissue engineering approaches.

In this review, we will highlight current progress and emerging approaches in cell manufacturing for cell-based tissue engineering, with a focus on vascularized, skeletal muscle regeneration. We first provide an overview of U.S. Food and Drug Administration (FDA) regulation of cell manufacturing for regenerative medicine and tissue engineering products. We then provide state-of-the-art approaches for manufacturing endothelial cells and muscle stem cells for tissue engineering and regenerative medicine; these current approaches focus on maintaining the identity and potency of the cells while exponentially expanding the cells. We then discuss emerging approaches in cell manufacturing that will be critical for measuring and manipulating cellular heterogeneity in engineered tissues and organs.

### U.S. FDA regulation of cell manufacturing and tissue-engineered products

As the primary governmental body regulating medical therapies in the United States, the FDA is responsible for the approval and clearance of cell therapies and biomedical devices, including tissue engineering therapies. The FDA works toward ensuring the safety and efficacy of new therapies before they are marketed as products available for public use. The FDA mediates the regulation of regenerative medicine products in the U.S. via the Food, Drug, and Cosmetics Act (FD&C), the Public Health Service Act, and additional guidelines (Center for Biologics Evaluation and Research, 2019). This collection of acts is helpfully surmised in the Compliance Policy Guides (CPGs) responsible for each product category. Regenerative medicine technology classification varies depending on the exact technology used, and the novel nature of these products often complicates accurate classification.

Cellular products, tissues, and their derivatives are regulated under the human cell, tissue, and cellular and tissue-based product (HCT/P) category, belonging to the larger scope of vaccines, bloods, and biologics (CFR Chapter I Subchapter F Biologics, 2025) under the Center for Biologics and Evaluation Research (CBER) at the FDA. If the products are unmanipulated, vascularized tissues, then the products are regulated by the Health Resources and Services Administration; tissue engineered products that are vascularized *in vitro* do not fall under this designation. Combination products for regenerative medicine may also fall under the category of medical devices within the Center for Devices and Radiological Health (CDRH). These combination products may include the delivery of manufactured cells combined with some form of extracellular matrix or scaffold (either man-made or naturally derived). These combination products must comply with all safety standards of their respective components in addition to the combinatory product itself, as dictated in the Code of Federal Regulations (21 CFR Part 4 -- Regulation of Combination Products, no date). These regulations also apply to the delivery of growth factors, free molecules, and other therapeutics from devices in situ.

Recognizing the challenges of seeking and obtaining regulatory approval, the FDA and similar regulatory agencies have created special programs for tissue products to streamline this process. Acknowledging the ever-increasing demand for the evaluation and approval of newer tissue developments, CBER created the Office of Therapeutic Products (OTP) in 2023. OTP evaluates new cell and gene therapy products to determine if their safety and benefits are worth the potential risks associated with human use. The more distinct from native human cells/tissue or the degree of novelty that the product has, the more complex the approval process will be, as these new products cannot rely on pre-existing approved technologies (Center for Biologics Evaluation and Research, 2022). If a product is shown to improve the clinical outcome of a preexisting treatment and/or decrease the side effects such that a patient may resume treatment that they would not otherwise be a candidate for, fast-track approval may be granted (Public Law 112 – 144, 2012). As of 2025, there are 45 approved cellular and gene therapy products for clinical use; out of those approved, 32 utilize cell therapies (Supplementary Table S1), and only 4 can be considered cell-based tissue engineering therapies (Table 1).

Part of the approval process for engineered tissues consists of Chemistry Manufacturing and Controls (CMC) guidance and regulations that ensure that the manufactured cell and biomaterial products are safe and potent for human utilization. Cytotoxicity, sterility, viability, and biocompatibility are a few of the basic requirements that apply to products regulated by OTP. In addition to these safety requirements, the FDA issues further guidance on the efficacy of products that fall under a Biologics License. These guidance include requirements for a cell product’s potency, which is defined as “the specific ability or capacity of the product, as indicated by appropriate laboratory tests or by adequately controlled clinical data obtained through the administration of the product in the manner intended, to effect a given result” (CFR Part 610 General Biological Products Standards, 2025). This guidance specifies that potency must be ascertained at the moment of application and in all subsequent lots during the manufacturing process. While the FDA does not explicitly dictate which tests must be performed to make this determination, it does offer guidance in developing these tests (Center for Biologics Evaluation and Research, 2020), and that the manufacturer must follow “current good manufacturing practices” in doing so (Center for Biologics Evaluation and Research, 2023). In the space of cell manufacturing, identity and potency determination is exceedingly important, as some processes may alter the original cells so that they are not classified as HCT/Ps via surface modifications, genetic engineering, or delivery in alternative methods (CFR 1.78 Biological products, 2025). The FDA has yet to determine the requirements for cell heterogeneity, except for those involving the purity of the sample concerning the presence of extraneous materials that are not required or resulting from a particular manufacturing process (CFR 610.13 Purity, 2025).

Recent changes to guidelines within the governing bodies, particularly for emerging technologies targeting diseases of great significance for the medical community and other similar programs, allow for more streamlined pathways from the benchtop to clinical trials, including those associated with cell manufacturing. Signed into law in 2016, the 21st Century Cures Act (CURES) provides the FDA and the larger community with new pathways for regenerative and medical device approval (Bonamici, 2016). Part of the innovation comes from using Real World Data/Evidence (RWD/E) as part of the considerations for approval (Office of the Commissioner, 2024). For regenerative medicine approaches, the product designation of a Regenerative Medicine Advanced Therapy (RMAT) would grant a faster approval process. This designation includes products such as cell therapies, therapeutic tissue engineering products, human cell and tissue products, or any combination product using such therapies or products (Bonamici, 2016; CBER, 2017). If a new product meets the definition of an RMAT, targets a life-threatening condition, and RWD shows that the product meets an unmet need for this disease, then this product is eligible for expedited procedures. This RMAT application would be filed in addition to the Investigational New Drug (IND) application.

Overall, these programs may expedite approval pathways for ongoing and existing interventional clinical trials funded by US Federal agencies and/or industry that are classified as “Regenerative Medicine”. For example, on-going clinical trials that focus on the usage of autologous cell therapies and allograft tissue samples for the treatment of joint-related diseases (R3 Stem Cell, 2021; Red de Terapia Celular, 2023; Healeon Medical Inc, 2024; InGeneronInc, 2024) can benefit from accelerated pathways such as the HCT/P, due to possibly being considered minimally processed human tissue/cells. Similarly, orthodontic and ischemic treatments that involve the delivery of targeted cells and cell-scaffold constructs (Neotherix Limited, 2017; Brizuel, 2020; Davis, 2021) can utilize other pathways under the CARES Act, such as those used in conjunction with a current therapy, to improve the current standard-of-care in the case of the ischemia treatments or as a treatment to a disease of interest of the FDA. Ultimately, these pathways can lead to early interactions between the FDA and sponsors, as well as mediate an accelerated approval process through previously approved surrogate/intermediate endpoints and data from a limited (but meaningful) number of peer-reviewed sources.

### State-of-the-art approaches for cell manufacturing for vascularized, skeletal muscle tissue engineering and regeneration

**FIGURE 1:**
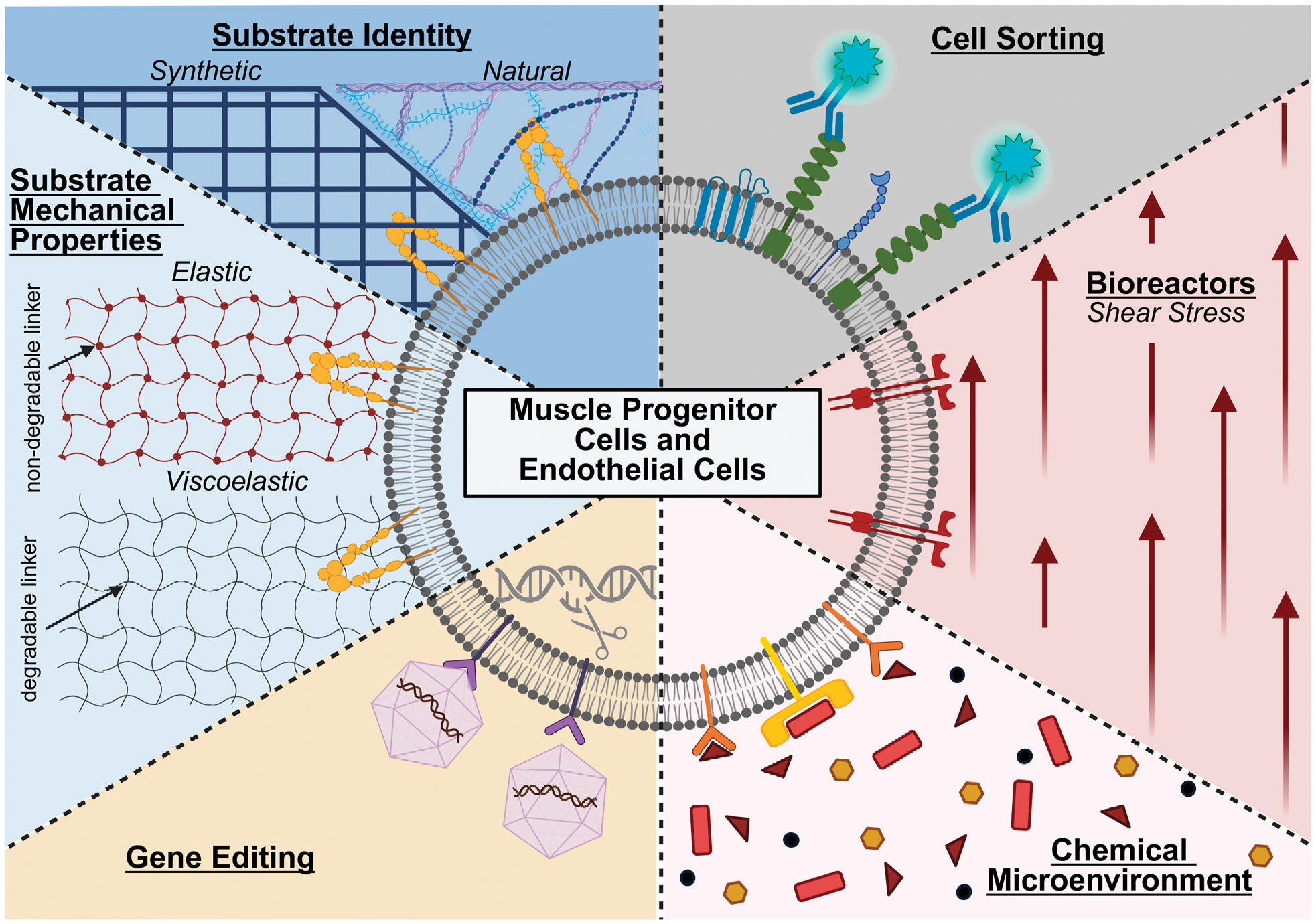
State-of-the-art approaches for manufacturing muscle progenitor cells and endothelial cells, including the use or modification of cell sorting, bioreactors, chemical microenvironments, gene editing, substrate mechanical properties, and substrate identity. Created in BioRender. (2025) https://BioRender.com/ogdvpr5.

State-of-the-art approaches for cell manufacturing, including those for endothelial cells and muscle stem cells, can enrich for particular cell populations, provide distinct chemical and physical microenvironments, and genetically alter cells’ phenotype and function (Figure 1). Often, these approaches are leveraged either separately or in combination during cell expansion to maintain cell identity and improve cellular potency. Moreover, these approaches require tight regulation to enable the cells’ ability to promote vascular and muscle regeneration. Careful consideration is also needed when choosing methods for cell manufacturing, as there are different advantages and disadvantages for each approach (Table 2).

### Cell sorting

Isolating and identifying cell populations are the first essential steps for manufacturing cells for cell-based tissue engineering approaches. Cell sorting is critical for the isolation and identification of cell populations and subpopulations with optimal cellular potency. Traditionally, endothelial cells and muscle satellite cells are isolated and sorted by distinct surface markers via label-based approaches, which involve tagging targets of interest with antibodies. The standard approach for isolating muscle satellite cells is to negatively select for non-myogenic cells (i.e., sort for CD31, CD45, Sca-1 negative cells) and positively select for certain markers (e.g., sort for α7-integrin positive cells) (Motohashi et al., 2014; Sincennes et al., 2017). Likewise, to isolate endothelial cells, immune and epithelial cells are depleted (i.e., remove CD45 and EpCAM cells), and endothelial cells are enriched (e.g., select for CD31 + cells) (Conchinha et al., 2021).

These label-based approaches are also commonly leveraged to identify subpopulations of cells with distinct functions. For example, single-cell RNA-seq of human satellite cells has identified subpopulations of cells, including CAV1^+^ cells, that are morphologically distinct and demonstrated enhanced cell engraftment upon transplantation (Barruet et al., 2020). Additionally, slow-dividing satellite cells isolated from mouse extensor digitorum longus muscle (identified by dilution of the membrane dye PKH26) demonstrated long-term self-renewal and enhanced contribution to muscle regeneration *in vivo* (Ono et al., 2012). Differences in the performance of subpopulations of endothelial cells have also been identified. In particular, endothelial cells with high CD34 expression have shown higher levels of IL-33 and Angiopoietin 2 production, which regulates their ability to induce Treg proliferation (Arakelian et al., 2023). These differences in subpopulation performance also extend to endothelial cell interactions with synthetic tissue-engineered vascular grafts, where cells with low FLRT2 expression have been shown to have a greater ability to adhere to the synthetic material (Wolfe et al., 2024).

However, these label-based approaches are limited by their cost and the persistence of sorting surface antibodies that can interfere with subsequent cell function, analysis, or testing. Label-free approaches, while less common, alleviate these limitations since they do not require antibody use. For example, label-free-inertial separation in microfluidic devices is increasingly utilized to isolate cell populations and have enriched for myogenic cells from a mixed population with larger fibroblast progenitors (Syverud et al., 2018). Unfortunately, label-free approaches for muscle and endothelial cells are limited and current approaches are low throughput; further development is needed before they are a practical and cost-effective alternative.

### Measuring cellular potency

Obtaining the desired cell composition is often not sufficient to guarantee the efficacy of regenerative medicine and tissue engineering therapies. Measuring cell potency during manufacturing processes is critical for ensuring clinical success of the therapy. Moreover, the method of determining cell potency depends on the cell type being analyzed. Endothelial cell and endothelial progenitor cell potency is often characterized by the cells’ ability to form new blood vessel networks, whether it be *de novo* vessel formation (vasculogenesis) or vessel formation from pre-existing vessels (angiogenesis). Traditionally, the potency of these cells has been measured via intracellular markers, such as the expression of nitric oxide synthase (eNOS) and pro-angiogenic markers (VEGF, HGF, IGF-1) (Bouloumié et al., 1999; Sekiguchi et al., 2009). Alternatively, endothelial cell potency can be functionally measured by their *in vitro* capacity to form 3D vasculature structures of distinct architecture in physiologically relevant microenvironments. Measured geometric metrics of the formed vasculature, such as mean vessel length, number of junctions, and number of endpoints (van der Schaft et al., 2004; Zudaire et al., 2011) indicate a more actively regenerating phenotype.

In the case of satellite cells and muscle progenitor cells (i.e., myoblasts) that fuse into multinucleated myofibers (precursors of muscle fibers), a higher fusion index, number of nuclei per myofiber, presence of myosin heavy chain, and increased expression of CD56 cellular markers indicate high differentiation potency (Joulia et al., 2003; Shefer et al., 2006; Thurner et al., 2018). Moreover, muscle satellite cell potency is often measured by the cells’ *in vivo* engraftment potency in muscle injury models. However, the regular use of *in vivo* potency assays may not be practical. In the future, a shift to *in vitro* 3D physiologically relevant myogenic potency assays may be necessary.

### Controlling culture conditions with bioreactors

Bioreactors control environmental conditions and allow for both dynamic cell culture and large-scale expansion of cells. These systems can control a variety of factors, including flow, nutrient supply, and loading of engineered cells and/or tissues. Shear stress, for example, has been shown to enhance the ability of iPSCs to mature into endothelial cells of an arterial-like phenotype (Sivarapatna et al., 2015). A more recent study has evaluated how different shear rates can influence the arterial markers NOTCH1 and EphrinB2 of human pluripotent stem cell-derived endothelial cells (Masumura et al., 2009).

Unfortunately, large-scale expansion of cells in simple flow bioreactors can be limited due to space. Thus, the use of hollow fibers and microcarriers in perfusion or stirred tank bioreactors have become increasingly utilized, as they increase culture surface area (Stephenson and Grayson, 2018; García-Fernández et al., 2020). In particular, the combination of 3D porous gelatin microcarriers and spinner flasks or stirred tank bioreactors has been shown to greatly improve long-term culture and large-scale expansion of muscle satellite cells and myoblasts (Liu et al., 2022). For example, bovine satellite cells demonstrated the ability to expand on CytoDEX microcarriers in a bench-top stir tank bioreactor over 38 days while maintaining their satellite cell phenotype via expression of Pax7 (Tzimorotas et al., 2023). However, future work will be necessary to overcome current limitations of bioreactors, which include contaminations due to complex platform design, difficulty monitoring and/or harvesting manufactured cells, complications with maintaining specific shear stresses, and overall cost. Each bioreactor design is susceptible to varying limitations based on the platform.

### Defined chemical microenvironments

Distinct combinations of chemical factors and proteins have been shown to be critical to the large-scale expansion of regenerative muscle and endothelial cells while maintaining cellular potency *in vitro* and *in vivo*. For example, the growth factors EGF, bFGF, and HGF are critical for myoblasts’ proliferation, morphology, and fusion potential compared to commercially available media (SKGM-2) only containing hEGF (Jarocha et al., 2014). Moreover, vitamins and minerals, such as L-Ascorbic acid, also increase muscle stem cell proliferation while maintaining the cells’ ability to differentiate (Zhu et al., 2022). For umbilical cord blood-derived endothelial colony-forming cells, human platelet lysates have been shown to increase the viability, reduce the apoptosis, and increase the proliferation of the cells on a variety of different substrates (Denecke et al., 2015).

Improvements in the *in vitro* expansion of muscle stem cells with defined chemical cues have also been shown to enhance the cells’ engraftment *in vivo*. T-cell derived inflammatory cytokines, including IL-1α, IL-13, TNF-α, and IFN-γ, were shown to potently induce long-term expansion of muscle satellite cells *in vitro* over 20 passages. These cytokines also contribute to muscle regeneration *in vivo,* by improving continuous repair of muscle following multiple rounds of muscle injury (Fu et al., 2015). The combination of biochemical and biophysical cues, including a small-molecule inhibitor of p38α/p38β mitogen-activated protein kinase, and soft polyethylene glycol hydrogels, allowed for the expansion of functional muscle stem cells from aged mice. These functional muscle stem cells can be used to regenerate damaged muscle in an aged mouse population (Cosgrove et al., 2014). Overall, these chemical factors and proteins offer a promising method for maintaining cellular potency. However, their use can be limited due to the high cost of these materials and recombinant proteins, especially as depleted factors and proteins need replenished in culture.

### Gene editing

Many gene editing approaches can be used for cell-based therapies to enhance cell function and potency, including zinc-finger proteins, transcription activator-like effector nucleases, and CRISPR-Cas9 (Ashmore-Harris and Fruhwirth, 2020). Particularly, gene editing approaches have been shown to improve cell function for regenerative medicine applications. For example, retroviral vectors have been used to induce insulin-like growth factor-I gene expression in myoblast cells to improve the contractile response of tissue constructs that the cells form (Sato et al., 2011). Furthermore, CRISPR-Cas9 gene editing is becoming a promising method for enhancing cellular potency. For example, CRISPR can confer certain advantages to cultured endothelial cells, such as resistance to TNF-α cytotoxicity via ablation of NLRX1 (Cai et al., 2019) and enhanced junctional integrity of formed blood vessels via ablation of the cytoplasmic domain of PECAM-1 (Liao et al., 2018). CRISPR/Cas9 has also been utilized to ablate class I major histocompatibility complex molecules, which allows endothelial cells to avoid activation of allogenic natural killer cells (Merola et al., 2019). The advantages of CRISPR-Cas9 have also extended to enhancing the potency of muscle progenitor cells. Specifically, dystrophin defects in iPSCs derived from patients with Duchenne muscular dystrophy were reversed with CRISPR-Cas9, resulting in normal myoblasts from the diseased patients (Young et al., 2016). Upregulation of IGF-1 expression with CRISPR-Cas9 in myoblasts also enhanced their differentiation and reduced DMSO-induced atrophy *in vitro* (Roberston et al., 2020). While CRISPR-Cas9 offers a promising method for cell manufacturing, it can be hindered by its high cost and low transfection efficiencies in primary cells. Furthermore, gene-editing technologies broadly may have off-target effects characterized by unintended edits outside of the target area.

### Substrate identity and dimensionality

The identity of the biomaterial substrate that regenerative cells are cultured on is a key determinant of their cellular potency during long-term expansion. These substrates, which include both synthetic and natural biomaterials, have been shown to be advantageous over traditional tissue culture polystyrene. Endothelial cells derived from pluripotent stem cells demonstrated greater expression of genes related to vessel development, ECM, and glycolysis when cultured in 3D thermoreversible PNIPAAM-Peg hydrogels compared to cultures on 2D Matrigel-coated plates (Lin et al., 2018). Natural polymers have also been utilized when manufacturing human pluripotent stem cell-derived endothelial cells in 3D alginate hydrogel tubes, which demonstrated higher expression of endothelial cell-related genes and rates of glycolysis (Lin et al., 2019). More recently, food-grade microcarriers made of collagen and eggshell membrane were shown to expand muscle stem cells and induce activated, proliferating muscle stem cells with altered cell adhesion patterns compared to stem cells expanded on conventional CytoDEX microcarriers (Andreassen et al., 2022). While natural materials have shown beneficial results for endothelial and muscle cells, reproducibility of natural substrates can be difficult due to the batch-to-batch variability. Monitoring of substrate variability when utilizing these types of substrates for cell manufacturing is necessary to ensure expected outcomes.

In addition to the identity of the cell substrate, specific cell culture surface modifications have been shown to improve cell morphology and proliferation while maintaining potency. Endothelial cells cultured on a matrix composed of fibrin, fibronectin, gelatin, and VEGF maintained greater proliferation potential and lower thrombogenic characteristics than endothelial cells cultured on gelatin after several passages (Prasad Chennazhy and Krishnan, 2005). Likewise, the expansion of mouse skeletal muscle progenitor cells on Matrigel-coated dishes resulted in higher proliferation and multinucleated myotubes compared to collagen-coated dishes (Shahini et al., 2018).

### Substrate mechanical properties

The mechanical properties of substrates can greatly affect cell growth and differentiation during cell expansion. The design of biomaterial mechanical properties can be fine-tuned to improve cell potency and culture. In particular, substrate stiffness has been extensively explored as a key parameter for cell expansion. For example, endothelial cells derived from various sources (umbilical vein, aorta, saphenous vein, and dermal microvasculature) demonstrated heterogeneous responses to hydrogel stiffness in terms of cell attachment, spreading, elongation, and proliferation (Wood et al., 2011). This is further illustrated for muscle progenitor cells, where soft hydrogels (12 kPa) improved cell potency, myoblast self-renewal *in vitro,* and muscle regeneration *in vivo* compared to culture on hard TCPS (10^6^ kPa) (Gilbert et al., 2010).

Furthermore, the viscoelasticity or elasticity of the material is critical to cell function and expansion. While not yet evaluated in the context of cell manufacturing, endothelial cells have been shown to be responsive to material viscoelasticity and plasticity in terms of their morphogenesis and proliferation (Shayan et al., 2023; Wei et al., 2023). Similar results have been observed for muscle progenitor cells. Specifically, an elastic chitosan/beta-glycerophosphate/collagen hydrogel mimicking the elastic modulus of muscle was used to expand myoblasts with greater proliferation capability, cell viability, colony-forming frequency, and potential for myogenic differentiation compared to myoblasts cultured on TCPS (Ding et al., 2015). The viscoelasticity and stress relaxation of the gel substrate have also been shown to greatly affect the proliferation and spreading of myoblasts. Namely, the stress-relaxing substrates resulted in greater spreading and proliferation of myoblasts compared to purely elastic substrates (Bauer et al., 2017). A significant hurdle when altering substrate mechanical properties is the ability to independently tune different mechanical properties of the materials, such as stiffness from viscoelasticity, which may be difficult based on the cross-linking chemistries of the material. This may inhibit the ability to match the native mechanical properties of certain *in vivo* tissues.

Across these diverse manufacturing methods, there are several existing limitations in manufacturing therapeutically potent cells for clinically relevant regenerative medicine therapies. For example, it remains a critical challenge to accurately recapitulate the complex chemical and physical microenvironment of native tissues during cell manufacturing protocols, which are likely required to maintain or improve cellular potency during *in vitro* expansion. Furthermore, there are significant hurdles in scaling the exponential expansion of these cell manufacturing approaches to meet the cell number and density needs of clinically sized, large-scale tissue-engineered constructs. For engineering tissues of high cellular density, such as skeletal muscle tissue, the manufacturing costs associated with producing large quantities cells (on the order of billions of cells) may make these engineered constructs prohibitively expensive. Furthermore, there is also significant variability in cell function across these diverse methods, further warranting improved standards and metrics for evaluating the function, potency, and *in vivo* integration of the cells and their resulting engineered constructs.

### Emerging approaches and future directions for manufacturing heterogeneous cells and tissues

#### Measuring cellular heterogeneity

Living tissues are composed of a multitude of different cell types. Even within the same cell population of an organ, research has shown the existence of different clusters of differentiation or specialization (Rodor et al., 2022). To engineer and regenerate tissues that match the form and function of our native tissues, there is a need for improved approaches to characterize the properties of these cell subgroups to reproduce spatial tissue heterogeneity. To measure this heterogeneity for cell manufacturing and tissue engineering, emerging omics technologies, including single-cell RNA sequencing (scRNA-seq), single-cell spatial transcriptomics, and single-cell mass cytometry, can be leveraged. These approaches provide single-cell level resolution of individual cells’ gene expression and protein production, which can be used to infer information about their identity. Ultimately, omics technologies may broadly be useful in evaluating whether manufactured cells maintain their intrinsic cell variation both during cell manufacturing processes and after forming 3D tissues relative to their native source tissues and organs.

Single-cell RNAseq (scRNA-Seq) is a critical technology in next-generation sequencing that analyzes individual cell transcriptomes. The technique, first developed in 2009 (Tang et al., 2009), was an expansion of earlier techniques, namely, bulk-RNA sequencing, that allowed for the measurement of all actively synthesized proteins within a population. These approaches have been applied to endothelial cells and muscle stem cells *in vivo* and *in vitro*. For example, scRNA-seq has been used to measure the heterogeneity of organ-specific endothelial cell populations, which uncovered 13 transcriptomically distinct endothelial cell subpopulations independent of tissue origin (Paik et al., 2020). In addition to identifying different clusters of endothelial cells, scRNA-seq has identified 2 clusters of cultured corneal endothelial cells that expressed increased expression of the functional markers ALCAM and CDH2, identifying these subpopulations as cells with high therapeutic potential (Català et al., 2023).

In the context of myogenic cells, scRNA-seq has identified 17 different genetic clusters in myoblasts extracted from adult muscle tissue, all of which had differentially expressed surface markers and intracellular proteins. This analysis identified heterogeneity in the cells regarding muscular cell differentiation and ultimate cell fate, with specialized subpopulations that expressed unique signatures in terms of senescence, satellite cell quiescence, anti-inflammatory activity, and oxidative stress phenotypes. Of note, these differences were not associated with sex or age in mice and human populations (Barruet et al., 2020).

In the field of vascular tissue engineering, scRNA-seq has also been adopted to characterize heterogeneities in engineered vasculature *in vitro* and *in vivo*. Identification of the heterogeneous cellular composition of the tissues can provide insights into tissue health and whether the tissues replicate key functions observed *in vivo*. For example, in engineered microvasculature, sc-RNAseq identified heterogeneities in endothelial cells cultured in 3D engineered microvessels with either straight or spiral geometry under different flow regimens. This analysis revealed that flow in spiral vessels induced a unique subpopulation of endothelial cells not found in straight vessels, which modified gene expression related to angiogenesis, vascular growth, and inflammatory stress responses (Mandrycky et al., 2020). scRNA-seq was also utilized to validate a personalized tissue-engineered vein (P-TEV) derived from decellularized allogenic vena cava grafts that were reconditioned with autologous peripheral blood vessels in a pig model. The analysis revealed that post-implantation, the regenerated vena cava from the P-TEV exhibited single-cell gene expression profiles similar to native vena cava, highlighting the success of the approach in recapitulating native tissue heterogeneity (Österberg et al., 2023).

In the context of skeletal muscle tissue engineering, sc-RNAseq has validated the heterogeneity of satellite cells and myoblasts within engineered muscular tissue. Recently, scRNA-seq was utilized to identify both quiescent and activated satellite cell subpopulations within engineered 3D human myofibers (Wang et al., 2022). In a similar engineered muscle from human induced pluripotent stem cells, scRNA-seq analysis revealed cell subpopulations modeling muscle, mesenchyme, and neural lineage, demonstrating successful emulation of important neural muscle developmental stages in 2D and 3D microenvironments (Shahriyari et al., 2022).

Where scRNA-seq provides single-cell level analysis of protein expression, spatial transcriptomics indicates the distribution of one or multiple genes. These genes are labeled to determine the relative location of gene expression in relation to other cell types. The trade-off and limitation with this spatial information compared to scRNA-seq is that fewer genes can be analyzed at a time on a cell-to-cell basis (Ståhl et al., 2016). These analyses have thus far been applied to native or graft vasculature and muscle tissue *in vivo* in preclinical models. For example, spatial transcriptomics was used to identify the spatial architecture of activated cells in vein grafts following distension during harvesting, thus highlighting specific regions and subpopulations of cells that respond to distension (Michaud et al., 2024). Furthermore, spatial transcriptomics was used to measure diversity in endothelial cells from different organs in diabetic patients as compared to healthy individuals (Wang et al., 2024). Likewise, the same technology was used to identify the disease markers in histological samples for Duchenne Muscular Dystrophy and Muscular Sarcoidosis (Heezen et al., 2023; Larouche et al., 2023; Lequain et al., 2023). There is significant potential for using these approaches to characterize engineered tissues, measure cellular dispersion within biomaterials, and ultimately utilize this information for product quality control. By better characterizing spatial cellular identity and heterogeneity, it is possible to obtain insights into how cell populations are seeded throughout biomaterials or injury sites.

Lastly, mass cytometry is a combination of two previously used techniques: flow cytometry and mass spectroscopy. Cells are fixed and labeled using antibodies conjugated to heavy metal isotopes, which are then run through the traditional mass spectroscopy process to identify them. This approach benefits from the high resolution, multiplexed, high throughput capabilities of mass spectroscopy without the shortfalls of traditional fluorescence flow cytometry, such as signal decay and spectral overlap. However, it does have challenges, including sample disintegration and low sensitivity (Spitzer and Nolan, 2016). Through Imaging Mass Cytometry (IMC), a form of mass cytometry that adds a high-fidelity, image scan of the sample, it is possible to obtain single-cell spatial transcriptomics resolution with a marker count like scRNA-seq. These approaches have thus found utility for identifying cell diversity for fundamental studies in regeneration and cancer. This includes identification of cells involved in pancreatic ductal adenocarcinoma, where endothelial cells were found to be closely associated with stromal cells and distantly separated from tumor and ductal cells (Sussman et al., 2024). In the context of muscle cells, these approaches can reconstruct *in vivo* cell heterogeneity data of the myeloid lineage gastrocnemius (GA) and tibialis anterior (TA) muscles (Porpiglia et al., 2017), as well as evaluate differentiation and autophagy of *in vitro* cultured myoblast cells (Brown et al., 2021). However, these methods are limited by their cost, throughput, sensitivity, and resolution. As mass cytometry gains more widespread adoption and becomes more cost-effective, these approaches will also become adopted for characterizing engineered tissues.

#### Future directions for manipulating cellular heterogeneity

**FIGURE 2:**
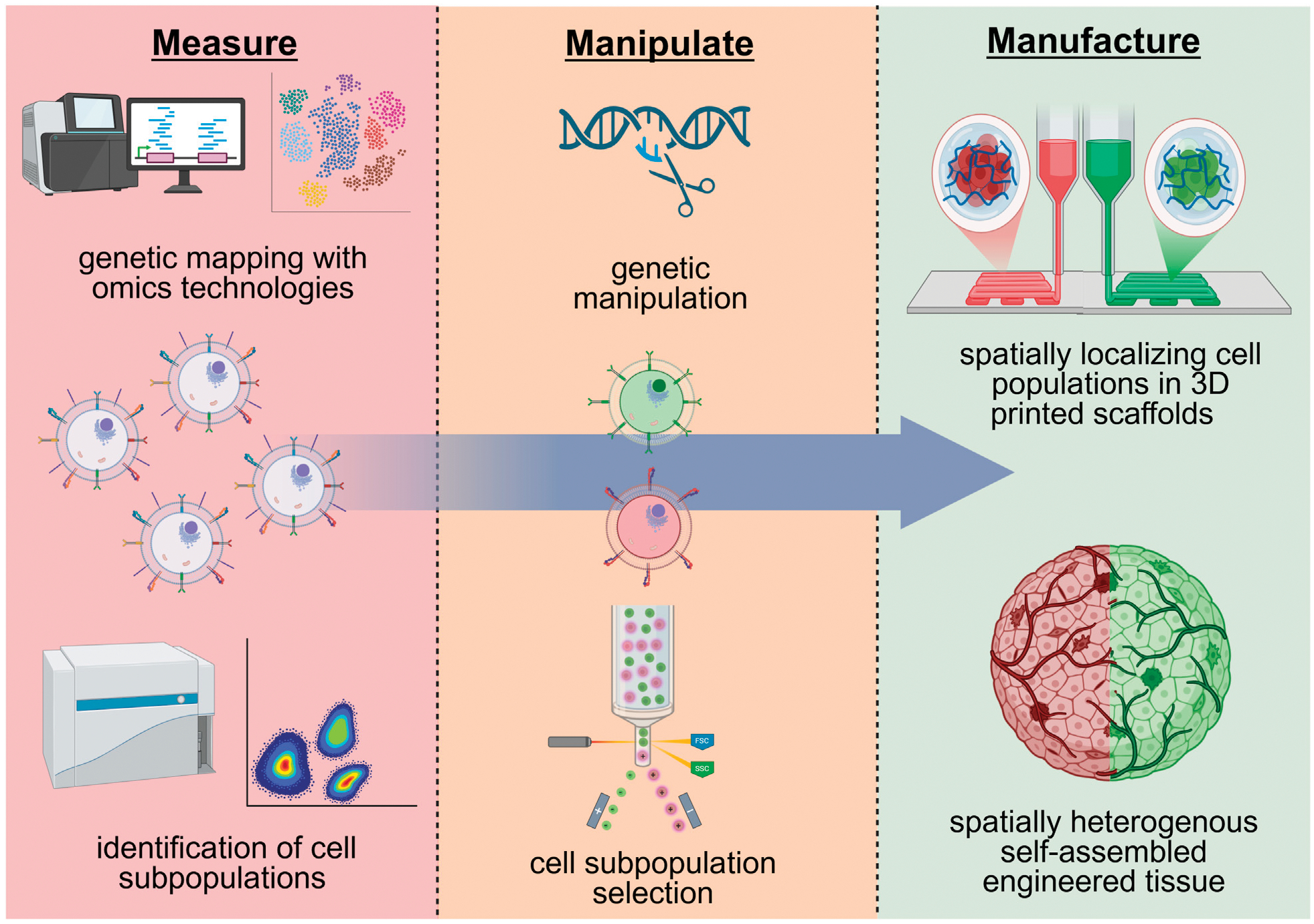
Potential future approaches for regenerative cell manufacturing that leverage and manipulate cell heterogeneity. A hypothetical approach could entail the identification of cell heterogeneity via genetic and phenotypical markers with omics technologies, altering these subpopulations via genetic engineering and/or cell sorting, and the spatial localization of heterogenous cell populations in 3D printed scaffolds. Created in BioRender. (2025) https://BioRender.com/ogdvpr5.

Insights from the aforementioned omics technologies can pave the way for future approaches to utilize cell heterogeneity to engineer tissues that model the form and function of our native tissues. By leveraging innovative biomanufacturing techniques, researchers can manipulate cell diversity to achieve three general outcomes: 1) selectively choose specific cellular populations that are associated with particular tissue-forming capacities and/or positive patient outcomes (i.e., removing cells associated with fibrosis and chronic inflammation); 2) recapitulate native tissue structure by tailoring the proportions of specific cell subpopulations to reconstruct the native proportions or to a more favorable ratio for regeneration; 3) spatially reconstruct cell positioning with the advent of additive manufacturing techniques. These outcomes can be achieved via specific approaches, such as cell sorting, genetic engineering, and 3D bioprinting, which can manipulate cell variations to further improve cell potency and cell spatial localization (Figure 2). Engineering spatially organized and controlled tissues with these approaches is critical, as disorganized or varied cell populations are associated with certain disease states (Wu et al., 2022).

A proper understanding of cell variability and subpopulations can be leveraged to enrich specific cell groups that exhibit specific functions and/or tissue-forming capacity (Figure 2). For example, cell sorting of unique endothelial cell subpopulations identified by sc-RNAseq (Paik et al., 2020; Mandrycky et al., 2020) may be used to enrich for cells that self-assemble in blood vessels of distinct geometries (diameter, branching point, length), permeability, and antigen-presentation capacity. In the context of muscle stem cells, cell sorting may select cells identified from scRNA-seq (Barruet et al., 2020; Lovrić et al., 2022) that form muscle fibers of distinct shape, type, and contractile function. These approaches would overall provide tissue engineers greater control over tissues formed by cellular self-assembly at injury sites or in biomaterials. The expression of integrins (Kwee et al., 2021) and other cell adhesion proteins, such as cell cadherins, may also be targets for sorting out these unique subpopulations due to their critical role in tissue self-assembly and extracellular matrix remodeling. This would overall improve upon existing approaches to engineer self-assembled tissues in biomaterials that often yield tissues with a disorganized and random distribution of tissue structures.

With improved understanding of transcriptomics and molecular mechanisms underlying diverse cell groups, genetic engineering may be used for the targeting of specific gene(s) to manipulate cell diversity and variation (Figure 2). Specifically, cells can be genetically modified to alter the expression of key surface markers or genes that are representative of distinct cellular subpopulations with a particular function or tissue-forming capacity. Building off previous work, for example, satellite cells may be genetically modified to upregulate CAV1 to match the phenotype of CAV1+ satellite cell subpopulations that demonstrate enhanced engraftment and muscle formation *in vivo* (Barruet et al., 2020). Similarly, endothelial cells may be genetically modified to upregulate CD34 to enhance paracrine signaling function, due to identified cell subpopulations of high CD34 expression have increased IL-33 and Angiopoietin 2 production (Arakelian et al., 2023). Modulation of integrin expression, which has been utilized to identify cells in biomaterials of distinct potency, may also be a target for genetic manipulation to regulate cell potency in biomaterial scaffolds (Kwee et al., 2021). These genetic engineering approaches may provide a means to generate and manufacture large quantities of cells of a distinct subpopulation when the subpopulation of cells is limited in number and cannot be expanded *in vitro*.

The emergence of various 3D printing technologies offers a variety of biomanufacturing approaches to manipulate and form spatially heterogeneous tissues. 3D printing has been utilized extensively to precisely engineer the architecture and placement of cells and biomaterials for skeletal muscle (Choi et al., 2016) and vascular tissue engineering (Kolesky et al., 2016). In these existing approaches, different materials of varying composition, fiber alignment, and mechanical properties have been spatially organized with 3D printing (Choi et al., 2016). Furthermore, 3D printing has allowed for complex, multiscale vessel networks and positioning of different cell types in appropriate locations (Kolesky et al., 2014; 2016). Approaches have also been developed that allow 3D printing of distinct growth factors throughout engineered scaffolds, particularly to spatially localize distinct regions of blood vessel and bone formation (Freeman et al., 2020).

Moving forward, the 3D bioprinting of subpopulations of cells with distinct tissue-forming capacity can be leveraged to precisely seed cells throughout different locations of a biomaterial scaffold to form heterogeneous self-assembled tissues (Figure 2). For example, endothelial cell subpopulations with unique vessel-forming capacity may be spatially distributed throughout biomaterials to form self-assembled vasculature with spatial hierarchy of vessel geometries. This may lead to distinct regions where arteries/veins, arterioles/venules, and capillaries are formed by cellular self-assembly. Furthermore, spatially distribution of muscle progenitor cells within biomaterials may lead to self-assembled muscle fibers of defined distribution in muscle fiber type and size. This will be critical for engineering muscles that have specific and controlled contractile function, as muscle structural heterogeneity is critical for muscles to strain and contract in specific orientations (Azizi and Deslauriers, 2014).

A critical future direction in leveraging and manipulating these cell heterogeneities will be the creation of standards and metrics for measuring tissue and organ level heterogeneity and function. The standard metric for evaluating the heterogeneity of engineered tissues and cultured cells is to compare scRNA-seq analysis of the cells to scRNA-seq analysis of *in vivo* tissues and organs as a semi-qualitative metric of matching native tissue diversity. Comparison of engineered tissue and native spatial transcriptomics are also utilized as a metric of structural tissue heterogeneity, but this is limited by the 2D nature of these analyses. There is a critical need for improved approaches to measure cellular and tissue-level heterogeneity in 3D tissue structures in a robust manner. Furthermore, tissue and organ specific standards to evaluate the long-term function and integration of the engineered constructs are critical to demonstrate that the tissues also match the physiological diversity of the tissues. This may include standards to evaluate the ability of muscle to contract in different orientations or blood vessels to anastomosis with the host vasculature and deliver nutrients with similar efficacy as their native counterparts. Further development in these areas in conjunction with the enhanced cell manufacturing approaches described in this review are necessary for the clinical translation of cell-based tissue engineering.

## Conclusion

Cell-based tissue engineering presents tremendous potential for regenerative medicine, but its clinical effectiveness is still limited due to the difficulties in producing regenerative cells that accurately mirror the complexity of native tissues. This review highlights how the FDA’s regulatory landscape is adapting to accommodate innovative therapies, yet a significant gap persists in recognizing both cellular variation and potency as critical design characteristics for engineering tissues. Current cell manufacturing methods for regenerative medicine tend to emphasize the expansion of generalized cell populations with high potency; however, these methods often overlook the fact that native tissues are defined by their tissue-specific, intricate cellular diversity and spatial arrangement.

In this review, we examined existing techniques for manufacturing endothelial cells and muscle stem cells, which are vital for developing vascularized skeletal muscle. Specifically, we have outlined contemporary methodologies for isolating, expanding, and enhancing the potency of endothelial cells and muscle stem cells, key players in constructing vascularized skeletal muscle. Moving forward, researchers and industry must focus on measuring, understanding, and manipulating single-cell heterogeneity. The rise of single-cell omics technologies, such as single-cell RNA sequencing, offers an opportunity to gain insights into the local and systemic impact of different cell subpopulations within tissues. To leverage this knowledge, future strategies for cell manufacturing should incorporate advanced sorting techniques to isolate specific, functionally relevant cell populations. Of note, emerging technologies that use specific surface marker targeting, such as integrins, as identifiers for cellular potency differentiators may have promising results in the creation of 3D microvasculature and myofiber formation. Additionally, 3D bioprinting of identified cellular subpopulations can direct their spatial arrangement and genetic engineering can modify intrinsic heterogeneities of cell populations. By intentionally integrating and managing cellular heterogeneity throughout the manufacturing process, we can bypass the limitations of creating homogeneous constructs. This approach would enable us to develop sophisticated tissue replacements that match the structural and functional complexities necessary for broader clinical adoption.

## Supplementary Material

Supplementary material

The Supplementary Material for this article can be found online at: https://www.frontiersin.org/articles/10.3389/fceng.2025.1637075/full#supplementary-material

## Figures and Tables

**TABLE 1:** List of approved cellular therapy products for tissue engineering from the Office of Therapeutic Products at the U.S. Food and Drug Administration (Current as of 1 July 2025).

Name	Manufacturer	Type	Usage	Components
LAVIV	Fibrocell Technologies	Patient Cells	Nasolabial Fold Wrinkles	Fibroblasts in Media
GINTUIT	Organogenesis	Encapsulated Cells	Mucogingival Conditions	Keratinocytes and Fibroblasts in Bovine Collagen
MACI	Vericel		Cartilage Defects	Chondrocytes in Porcine Collagen
STRATAGRAFT	Stratatech		Skin Burns	Keratinocytes and Fibroblasts in Murine Collagen

**TABLE 2:** A comparison of different cell manufacturing methods and categories.

Method	Description	Advantages	Disadvantages
Cell Sorting	Sorting of cells into distinct populations or subpopulations utilizing label-based and label-free approaches	Precisely identify and obtain certain (sub) populations of cells with distinct function	Surface antibodies of label-based approaches may interfere with cell function. Options for label-free sorting approaches are limited and are low throughput
Bioreactors	Systems that control dynamic culture conditions	Control of parameters and provide cells physiologically relevant microenvironment	Potentially limited visibility and monitoring of cells during culture. Cost depending on the system
Chemical Microenvironments	Use of chemical factors and proteins in culture	Potent chemical factors and proteins can easily be added to culture	Cost of chemical factors and recombinant proteins that need be replenished throughout culture
Gene Editing	Insertion, deletion, replacement, or modification of DNA	Can confer permanent or semi-permanent specific changes in cellular phenotype	Off-target effects, costs, efficiencies in primary cells
Substrate Identity and Dimensionality	Material type (natural vs synthetic) and modification of the material	Material type and modification can be fine-tuned	Batch to batch variability of natural materials
Substrate Mechanical Properties	Mechanical properties of the substrate including stiffness, viscoelasticity, or elasticity	Mechanical properties can be fine-tuned for most materials by changing material fabrication	Matching the mechanical properties of native tissue can be difficult based on material properties and cross-linking chemistries

## References

[R1] Andreassen RC, Rønning SB, Solberg NT, Grønlien KG, Kristoffersen KA, Høst V, (2022). Production of food-grade microcarriers based on by-products from the food industry to facilitate the expansion of bovine skeletal muscle satellite cells for cultured meat production. Biomaterials 286, 121602. doi:10.1016/j.biomaterials.2022.121602 35660866

[R2] Arakelian L, Lion J, Churlaud G, Bargui R, Thierry B, Mutabazi E, (2023). Endothelial CD34 expression and regulation of immune cell response *in-vitro*. Sci. Rep 13 (1), 13512. doi:10.1038/s41598-023-40622-7 37598252 PMC10439936

[R3] Ashmore-Harris C, and Fruhwirth GO (2020). The clinical potential of gene editing as a tool to engineer cell-based therapeutics. Clin. Transl. Med 9 (1), 15. doi:10.1186/s40169-020-0268-z 32034584 PMC7007464

[R4] Azizi E, and Deslauriers AR (2014). Regional heterogeneity in muscle fiber strain: the role of fiber architecture. Front. Physiology 5, 303. doi:10.3389/fphys.2014.00303 PMC4129366 25161626

[R5] Barruet E, Garcia SM, Striedinger K, Wu J, Lee S, Byrnes L, (2020). “Functionally heterogeneous human satellite cells identified by single cell RNA sequencing”. Editors Tajbakhsh S, Cheah KSE, and Olwin BB, 9. doi:10.7554/eLife.51576 PMC7164960 32234209

[R6] Bauer A, Gu L, Kwee B, Li WA, Dellacherie M, Celiz AD, (2017). Hydrogel substrate stress-relaxation regulates the spreading and proliferation of mouse myoblasts. Acta Biomater. 62, 82–90. doi:10.1016/j.actbio.2017.08.041 28864249 PMC5641979

[R7] Bonamici S (2016). 21st century cures act. Available online at: https://www.congress.gov/bill/114th-congress/house-bill/34.

[R8] Bouloumié A, Schini-Kerth VB, and Busse R (1999). Vascular endothelial growth factor up-regulates nitric oxide synthase expression in endothelial cells. Cardiovasc. Res 41 (3), 773–780. doi:10.1016/S0008-6363(98)00228-4 10435050

[R9] Brizuel DC (2020). Encapsulated mesenchymal stem cells for endodontic treatment of permanent teeth with apical lesion: a controlled clinical trial. Clinical trial registration NCT03102879. Clinicaltrials.gov. Available online at: https://clinicaltrials.gov/study/NCT03102879 (Accessed June 26, 2025).

[R10] Brown HMG, Kuhns MM, Maxwell Z, and Arriaga EA (2021). Nonspecific binding correction for single-cell mass cytometric analysis of autophagy and myoblast differentiation. Anal. Chem 93 (3), 1401–1408. doi:10.1021/acs.analchem.0c03211 33348978 PMC9487344

[R11] Cai M, Li S, Shuai Y, Li J, Tan J, and Zeng Q (2019). Genome-wide CRISPR-Cas9 viability screen reveals genes involved in TNF-α-induced apoptosis of human umbilical vein endothelial cells. J. Cell. Physiology 234 (6), 9184–9193. doi:10.1002/jcp.27595 30317623

[R12] Català P, Groen N, LaPointe VLS, and Dickman MM (2023). A single-cell RNA-Seq analysis unravels the heterogeneity of primary cultured human corneal endothelial cells. Sci. Rep 13 (1), 9361. doi:10.1038/s41598-023-36567-6 37291161 PMC10249941

[R13] CBER (2017). Expedited programs for regenerative medicine therapies for serious conditions. Washington, DC FDA. Available online at: https://downloads.regulations.gov/FDA-2017-D-6159-0017/attachment_1.pdf (Accessed: April 24, 2025).

[R14] Center for Biologics Evaluation and Research (2019). Potency tests for cellular and gene therapy products. Washington, DC FDA. Available online at: https://www.fda.gov/regulatory-information/search-fda-guidance-documents/potency-tests-cellular-and-gene-therapy-products (Accessed March 17, 2025).

[R15] Center for Biologics Evaluation and Research (2020). ‘Framework for the regulation of regenerative medicine products’, Available online at: https://www.fda.gov/vaccines-blood-biologics/cellular-gene-therapy-products/framework-regulation-regenerative-medicine-products (Accessed: 17 February 2025).

[R16] Center for Biologics Evaluation and Research (2022). Regulation of human cells, tissues, and cellular and tissue-based products (HCT/Ps) - small entity compliance guide. Washington, DC FDA. Available online at: https://www.fda.gov/regulatory-information/search-fda-guidance-documents/regulation-human-cells-tissues-and-cellular-and-tissue-based-products-hctps-small-entity-compliance (Accessed March 17, 2025).

[R17] Center for Biologics Evaluation and Research (2023). Potency assurance for cellular and gene therapy products. Washington, DC FDA. Available online at: https://www.fda.gov/regulatory-information/search-fda-guidance-documents/potency-assurance-cellular-and-gene-therapy-products (Accessed March 17, 2025).

[R18] CFR 1.78 Biological products (2025). CFR 1.78 -- biological products, HCT/Ps, and related drugs and medical devices Available online at: https://www.ecfr.gov/current/title-21/part-1/section-1.78 (Accessed March 17, 2025).

[R19] CFR 610.13 Purity (2025). CFR 610.13 -- purity Available online at: https://www.ecfr.gov/current/title-21/part-610/section-610.13 (Accessed March 17, 2025).

[R20] CFR Chapter I Subchapter F Biologics (2025). CFR chapter I subchapter F -- biologics. Available online at: https://www.ecfr.gov/current/title-21/chapter-I/subchapter-F (Accessed February 17, 2025).

[R21] CFR Part 4 Regulation of Combination Products (2025). CFR part 4 -- regulation of combination products. Available online at: https://www.ecfr.gov/current/title-21/part-4 (Accessed March 17, 2025).

[R22] CFR Part 610 General Biological Products Standards (2025). CFR part 610 -- general biological products standards. Available online at: https://www.ecfr.gov/current/title-21/part-610 (Accessed March 17, 2025).

[R23] Cho DS, and Doles JD (2017). Single cell transcriptome analysis of muscle satellite cells reveals widespread transcriptional heterogeneity. Gene 636, 54–63. doi:10.1016/j.gene.2017.09.014 28893664 PMC5659767

[R24] Choi Y-J, Kim TG, Jeong J, Yi H, Park JW, Hwang W, (2016). 3D cell printing of functional skeletal muscle constructs using skeletal muscle-derived bioink. Adv. Healthc. Mater 5 (20), 2636–2645. doi:10.1002/adhm.201600483 27529631

[R25] Conchinha NV, Sokol L, Teuwen LA, Veys K, Dumas SJ, Meta E, (2021). Protocols for endothelial cell isolation from mouse tissues: brain, choroid, lung, and muscle. Star. Protoc 2 (3), 100508. doi:10.1016/j.xpro.2021.100508 34585146 PMC8450255

[R26] Cosgrove BD, Gilbert PM, Porpiglia E, Mourkioti F, Lee SP, Corbel SY, (2014). Rejuvenation of the muscle stem cell population restores strength to injured aged muscles. Nat. Med 20 (3), 255–264. doi:10.1038/nm.3464 24531378 PMC3949152

[R27] Davis BR (2021). A phase II, randomized, placebo-controlled study of the safety, feasibility, and efficacy of autologous mesenchymal stem cells and C-kit+ cardiac stem cells, alone or in combination, administered transendocardially in subjects with ischemic HF. Clinical trial registration NCT02501811. Clinicaltrials.gov. Available online at: https://clinicaltrials.gov/study/NCT02501811 (Accessed June 26, 2025).

[R28] Denecke B, Horsch LD, Radtke S, Fischer JC, Horn PA, and Giebel B (2015). Human endothelial colony-forming cells expanded with an improved protocol are a useful endothelial cell source for scaffold-based tissue engineering. J. Tissue Eng. Regen. Med 9 (11), E84–E97. doi:10.1002/term.1673 23436759

[R29] Ding K, Yang Z, Xu J. z., Liu W. y., Zeng Q, Hou F, (2015). Elastic hydrogel substrate supports robust expansion of murine myoblasts and enhances their engraftment. Exp. Cell. Res 337 (1), 111–119. doi:10.1016/j.yexcr.2015.07.021 26210646

[R30] Dumas SJ, Meta E, Borri M, Luo Y, Li X, Rabelink TJ, (2021). Phenotypic diversity and metabolic specialization of renal endothelial cells. Nat. Rev. Nephrol 17 (7), 441–464. doi:10.1038/s41581-021-00411-9 33767431 PMC7993417

[R31] Freeman FE, Pitacco P, van Dommelen LHA, Nulty J, Browe DC, Shin JY, (2020). 3D bioprinting spatiotemporally defined patterns of growth factors to tightly control tissue regeneration. Sci. Adv 6 (33), eabb5093. doi:10.1126/sciadv.abb5093 32851179 PMC7428335

[R32] Fu X, Xiao J, Wei Y, Li S, Liu Y, Yin J, (2015). Combination of inflammation-related cytokines promotes long-term muscle stem cell expansion. Cell. Res 25 (6), 655–673. doi:10.1038/cr.2015.58 25976405 PMC4456625

[R33] García-Fernández C, López-Fernández A, Borrós S, Lecina M, and Vives J (2020). Strategies for large-scale expansion of clinical-grade human multipotent mesenchymal stromal cells. Biochem. Eng. J 159, 107601. doi:10.1016/j.bej.2020.107601

[R34] Gilbert P, Havenstrite KL, Magnusson KEG, Sacco A, Leonardi NA, Kraft P, (2010). Substrate elasticity regulates skeletal muscle stem cell self-renewal in culture. Sci. (New York, N.Y.) 329 (5995), 1078–1081. doi:10.1126/science.1191035 PMC2929271 20647425

[R35] Gunawardana H, Romero T, Yao N, Heidt S, Mulder A, Elashoff DA, (2021). Tissue-specific endothelial cell heterogeneity contributes to unequal inflammatory responses. Sci. Rep 11 (1), 1949. doi:10.1038/s41598-020-80102-w 33479269 PMC7820348

[R36] Healeon Medical Inc (2024). Adipose-derived biocellular regenerative therapy in treatment of osteoarthritis (OA) and associated connective tissue degeneration and pain. Clinical trial registration NCT04238143. Clinicaltrials.gov. Available online at: https://clinicaltrials.gov/study/NCT04238143 (Accessed June 26, 2025).

[R37] Heezen LGM, Abdelaal T, van Putten M, Aartsma-Rus A, Mahfouz A, and Spitali P (2023). Spatial transcriptomics reveal markers of histopathological changes in Duchenne muscular dystrophy mouse models. Nat. Commun 14 (1), 4909. doi:10.1038/s41467-023-40555-9 37582915 PMC10427630

[R38] InGeneron, Inc (2024). A longitudinal, multi-center safety study of autologous adult adipose-derived regenerative cell injection into chronic partial-thickness rotator cuff tears. Clinical trial registration NCT05400798. Clinicaltrials.gov. Available online at: https://clinicaltrials.gov/study/NCT05400798 (Accessed June 26, 2025).

[R39] Jarocha D, Stangel-Wojcikiewicz K, Basta A, and Majka M (2014). Efficient myoblast expansion for regenerative medicine use. Int. J. Mol. Med 34 (1), 83–91. doi:10.3892/ijmm.2014.1763 24788458 PMC4072397

[R40] Joulia D, Bernardi H, Garandel V, Rabenoelina F, Vernus B, and Cabello G (2003). Mechanisms involved in the inhibition of myoblast proliferation and differentiation by myostatin. Exp. Cell. Res 286 (2), 263–275. doi:10.1016/S0014-4827(03)00074-0 12749855

[R41] Kolesky DB, Homan KA, Skylar-Scott MA, and Lewis JA (2016). Three-dimensional bioprinting of thick vascularized tissues. Proc. Natl. Acad. Sci 113 (12), 3179–3184. doi:10.1073/pnas.1521342113 26951646 PMC4812707

[R42] Kolesky DB, Truby RL, Gladman AS, Busbee TA, Homan KA, and Lewis JA (2014). 3D bioprinting of vascularized, heterogeneous cell-laden tissue constructs. Adv. Mater 26 (19), 3124–3130. doi:10.1002/adma.201305506 24550124

[R43] Kwee BJ, Lam J, Akue A, KuKuruga MA, Zhang K, Gu L, (2021). Functional heterogeneity of IFN-γ–licensed mesenchymal stromal cell immunosuppressive capacity on biomaterials. Proc. Natl. Acad. Sci 118 (35), e2105972118. doi:10.1073/pnas.2105972118 34446555 PMC8536328

[R44] Larouche JA, Wallace EC, Spence BD, Buras E, and Aguilar CA (2023). Spatiotemporal mapping of immune and stem cell dysregulation after volumetric muscle loss. JCI Insight 8 (7). doi:10.1172/jci.insight.162835 PMC10132146 36821376

[R45] Lequain H, Dégletagne C, Streichenberger N, Valantin J, Simonet T, Schaeffer L, (2023). Spatial transcriptomics reveals signatures of histopathological changes in muscular sarcoidosis. Cells 12 (23), 2747. doi:10.3390/cells12232747 38067175 PMC10706822

[R46] Levenberg S, Rouwkema J, Macdonald M, Garfein ES, Kohane DS, Darland DC, (2005). Engineering vascularized skeletal muscle tissue. Nat. Biotechnol 23 (7), 879–884. doi:10.1038/nbt1109 15965465

[R47] Li Q, Zhu Z, Wang L, Lin Y, Fang H, Lei J, (2021). Single-cell transcriptome profiling reveals vascular endothelial cell heterogeneity in human skin. Theranostics 11 (13), 6461–6476. doi:10.7150/thno.54917 33995668 PMC8120211

[R48] Liao D, Mei H, Hu Y, Newman DK, and Newman PJ (2018). CRISPR-Mediated deletion of the PECAM-1 cytoplasmic domain increases receptor lateral mobility and strengthens endothelial cell junctional integrity. Life Sci. 193, 186–193. doi:10.1016/j.lfs.2017.11.002 29122551 PMC5754039

[R49] Lin H, Du Q, Li Q, Wang O, Wang Z, Elowsky C, (2019). Manufacturing human pluripotent stem cell derived endothelial cells in scalable and cell-friendly microenvironments. Biomaterials Sci. 7 (1), 373–388. doi:10.1039/C8BM01095A 30484784

[R50] Lin H, Du Q, Li Q, Wang O, Wang Z, Sahu N, (2018). A scalable and efficient bioprocess for manufacturing human pluripotent stem cell-derived endothelial cells. Stem Cell. Rep 11 (2), 454–469. doi:10.1016/j.stemcr.2018.07.001 PMC6092882 30078557

[R51] Liu Y, Wang R, Ding S, Deng L, Zhang Y, Li J, (2022). Engineered meatballs *via* scalable skeletal muscle cell expansion and modular micro-tissue assembly using porous gelatin micro-carriers. Biomaterials 287, 121615. doi:10.1016/j.biomaterials.2022.121615 35679644

[R52] Lovrić A, Rassolie A, Alam S, Mandić M, Saini A, Altun M, (2022). Single-cell sequencing deconvolutes cellular responses to exercise in human skeletal muscle. Commun. Biol 5 (1), 1121. doi:10.1038/s42003-022-04088-z 36273106 PMC9588010

[R53] Madden L, Juhas M, Kraus WE, Truskey GA, and Bursac N (2015). Bioengineered human myobundles mimic clinical responses of skeletal muscle to drugs. Elife. eLife Sci. Publ. Ltd 4, e04885. doi:10.7554/eLife.04885 PMC4337710 25575180

[R54] Mandrycky C, Hadland B, and Zheng Y (2020). 3D curvature-instructed endothelial flow response and tissue vascularization. Sci. Adv 6 (38), eabb3629. doi:10.1126/sciadv.abb3629 32938662 PMC7494348

[R55] Masumura T, Yamamoto K, Shimizu N, Obi S, and Ando J (2009). Shear stress increases expression of the arterial endothelial marker EphrinB2 in murine ES cells *via* the VEGF-notch signaling pathways. Arteriosclerosis, Thrombosis, Vasc. Biol 29 (12), 2125–2131. doi:10.1161/ATVBAHA.109.193185 19797707

[R56] Merola J, Reschke M, Pierce RW, Qin L, Spindler S, Baltazar T, (2019). Progenitor-derived human endothelial cells evade alloimmunity by CRISPR/Cas9-mediated complete ablation of MHC expression. JCI Insight 4 (20), e129739. doi:10.1172/jci.insight.129739 31527312 PMC6824302

[R57] Michaud ME, Mota L, Bakhtiari M, Thomas BE, Tomeo J, Pilcher W, (2024). Early injury landscape in vein harvest by single-cell and spatial transcriptomics. Circulation Res. 135 (1), 110–134. doi:10.1161/CIRCRESAHA.123.323939 38808504 PMC11189745

[R58] Mincarelli L, Lister A, Lipscombe J, and Macaulay IC (2018). Defining cell identity with single-cell omics. Proteomics 18 (18), 1700312. doi:10.1002/pmic.201700312 29644800 PMC6175476

[R59] Morgan JT, Shirazi J, Comber EM, Eschenburg C, and Gleghorn JP (2019). Fabrication of centimeter-scale and geometrically arbitrary vascular networks using *in vitro* self-assembly. Biomaterials 189, 37–47. doi:10.1016/j.biomaterials.2018.10.021 30384127 PMC6238648

[R60] Motohashi N, Asakura Y, and Asakura A (2014). Isolation, culture, and transplantation of muscle satellite cells. J. Vis. Exp. JoVE (86), 50846. doi:10.3791/50846 24747722 PMC4131689

[R61] Nakayama KH, Quarta M, Paine P, Alcazar C, Karakikes I, Garcia V, (2019). Treatment of volumetric muscle loss in mice using nanofibrillar scaffolds enhances vascular organization and integration. Commun. Biol 2 (1), 170–16. doi:10.1038/s42003-019-0416-4 31098403 PMC6505043

[R62] Neotherix Limited (2017). EktoTherix^™^ regenerative tissue scaffold for repair of surgical excision wounds. Clinical trial registration NCT02409628. Clinicaltrials.gov. Available online at: https://clinicaltrials.gov/study/NCT02409628 (Accessed June 26, 2025).

[R63] Office of the Commissioner (2024). Real-world evidence, FDA. FDA. Available online at: https://www.fda.gov/science-research/science-and-research-special-topics/real-world-evidence (Accessed: April 24, 2025).

[R64] Ono Y, Masuda S, Nam H. s., Benezra R, Miyagoe-Suzuki Y, and Takeda S (2012). Slow-dividing satellite cells retain long-term self-renewal ability in adult muscle. J. Cell. Sci 125 (5), 1309–1317. doi:10.1242/jcs.096198 22349695

[R65] Österberg K, Bogestål Y, Jenndahl L, Gustafsson-Hedberg T, Synnergren J, Holmgren G, (2023). Personalized tissue-engineered veins – long term safety, functionality and cellular transcriptome analysis in large animals. Biomaterials Sci. 11 (11), 3860–3877. doi:10.1039/D2BM02011D 37078624

[R66] Paik DT, Tian L, Williams IM, Rhee S, Zhang H, Liu C, (2020). Single-cell RNA sequencing unveils unique transcriptomic signatures of organ-specific endothelial cells. Circulation 142 (19), 1848–1862. doi:10.1161/CIRCULATIONAHA.119.041433 32929989 PMC7658053

[R67] Petrany MJ, Swoboda CO, Sun C, Chetal K, Chen X, Weirauch MT, (2020). Single-nucleus RNA-Seq identifies transcriptional heterogeneity in multinucleated skeletal myofibers. Nat. Commun 11 (1), 6374. doi:10.1038/s41467-020-20063-w 33311464 PMC7733460

[R68] Porpiglia E, Samusik N, Ho ATV, Cosgrove BD, Mai T, Davis KL, (2017). High-resolution myogenic lineage mapping by single-cell mass cytometry. Nat. Cell. Biol 19 (5), 558–567. doi:10.1038/ncb3507 28414312 PMC5728993

[R69] Prasad Chennazhy K, and Krishnan LK (2005). Effect of passage number and matrix characteristics on differentiation of endothelial cells cultured for tissue engineering. Biomaterials 26 (28), 5658–5667. doi:10.1016/j.biomaterials.2005.02.024 15878371

[R70] Public Law 112 – 144 (2012). Food Drug Adm. Saf. Innovation Act Available online at: https://www.govinfo.gov/app/details/PLAW-112publ144

[R71] Quarta M, Cromie M, Chacon R, Blonigan J, Garcia V, Akimenko I, (2017). Bioengineered constructs combined with exercise enhance stem cell-mediated treatment of volumetric muscle loss. Nat. Commun 8 (1), 15613. doi:10.1038/ncomms15613 28631758 PMC5481841

[R72] R3 Stem Cell (2021). Evaluation of regenerative medicine outcomes with umbilical allograft for musculoskeletal conditions. Clinical trial registration NCT03390920. Clinicaltrials.gov. Available online at: https://clinicaltrials.gov/study/NCT03390920 (Accessed June 26, 2025).

[R73] Rao L, Qian Y, Khodabukus A, Ribar T, and Bursac N (2018). Engineering human pluripotent stem cells into a functional skeletal muscle tissue. Nat. Commun 9 (1), 126. doi:10.1038/s41467-017-02636-4 29317646 PMC5760720

[R74] Red de Terapia Celular (2023). Treatment of lumbar degenerative disc disease with allogenic mesenchymal stem cells (MSV*) *MSV: bone marrow mesenchymal stromal cells expanded using the Valladolid IBGM procedure. Clinical trial registration NCT01860417. Clinicaltrials.gov. Available online at: https://clinicaltrials.gov/study/NCT01860417 (Accessed June 26, 2025).

[R75] Richter M, Piwocka O, Musielak M, Piotrowski I, Suchorska WM, and Trzeciak T (2021). From donor to the lab: a fascinating journey of primary cell lines. Front. Cell. Dev. Biol 9, 711381. doi:10.3389/fcell.2021.711381 34395440 PMC8356673

[R76] Roberston MJ, Raghunathan S, Potaman VN, Zhang F, Stewart MD, McConnell BK, (2020). CRISPR-Cas9–induced IGF1 gene activation as a tool for enhancing muscle differentiation *via* multiple isoform expression. FASEB J. 34 (1), 555–570. doi:10.1096/fj.201901107RR 31914652 PMC6956731

[R77] Rodor J, Chen SH, Scanlon JP, Monteiro JP, Caudrillier A, Sweta S, (2022). Single-cell RNA sequencing profiling of mouse endothelial cells in response to pulmonary arterial hypertension. Cardiovasc. Res 118 (11), 2519–2534. doi:10.1093/cvr/cvab296 34528097 PMC9400412

[R78] Sato M, Ito A, Kawabe Y, Nagamori E, and Kamihira M (2011). Enhanced contractile force generation by artificial skeletal muscle tissues using IGF-I gene-engineered myoblast cells. J. Biosci. Bioeng 112 (3), 273–278. doi:10.1016/j.jbiosc.2011.05.007 21646045

[R79] Schiaffino S, and Reggiani C (2011). Fiber types in Mammalian skeletal muscles. Physiol. Rev 91 (4), 1447–1531. doi:10.1152/physrev.00031.2010 22013216

[R80] Schoenfeld BJ, Vigotsky AD, Grgic J, Haun C, Contreras B, Delcastillo K, (2020). Do the anatomical and physiological properties of a muscle determine its adaptive response to different loading protocols? Physiol. Rep 8 (9), e14427. doi:10.14814/phy2.14427 32342648 PMC7186566

[R81] Sekiguchi H, Ii M, and Losordo DW (2009). The relative potency and safety of endothelial progenitor cells and unselected mononuclear cells for recovery from myocardial infarction and ischemia. J. Cell. Physiology 219 (2), 235–242. doi:10.1002/jcp.21672 19115244

[R82] Shahini A, Vydiam K, Choudhury D, Rajabian N, Nguyen T, Lei P, (2018). Efficient and high yield isolation of myoblasts from skeletal muscle. Stem Cell. Res 30, 122–129. doi:10.1016/j.scr.2018.05.017 29879622 PMC6090567

[R83] Shahriyari M, Islam MR, Sakib SM, Rinn M, Rika A, Krüger D, (2022). Engineered skeletal muscle recapitulates human muscle development, regeneration and dystrophy. J. Cachexia, Sarcopenia Muscle 13 (6), 3106–3121. doi:10.1002/jcsm.13094 36254806 PMC9745484

[R84] Shayan M, Huang MS, Navarro R, Chiang G, Hu C, Oropeza BP, (2023). Elastin-like protein hydrogels with controllable stress relaxation rate and stiffness modulate endothelial cell function. J. Biomed. Mater. Res. Part A 111 (7), 896–909. doi:10.1002/jbm.a.37520 PMC10159914 36861665

[R85] Shefer G, Van de Mark DP, Richardson JB, and Yablonka-Reuveni Z (2006). Satellite-cell pool size does matter: defining the myogenic potency of aging skeletal muscle. Dev. Biol 294 (1), 50–66. doi:10.1016/j.ydbio.2006.02.022 16554047 PMC2710453

[R86] Sincennes MC, Wang YX, and Rudnicki MA (2017). “Primary mouse myoblast purification using magnetic cell separation,” in Muscle stem cells: methods and protocols. Editors Perdiguero E, and Cornelison D New York, NY: Springer, 41–50. doi:10.1007/978-1-4939-6771-1_3 28247344

[R87] Singh VK, Saini A, Kalsan M, Kumar N, and Chandra R (2016). Describing the stem cell potency: the various methods of functional assessment and *in silico* diagnostics. Front. Cell. Dev. Biol 4, 134. doi:10.3389/fcell.2016.00134 27921030 PMC5118841

[R88] Sivarapatna A, Ghaedi M, Le AV, Mendez JJ, Qyang Y, and Niklason LE (2015). Arterial specification of endothelial cells derived from human induced pluripotent stem cells in a biomimetic flow bioreactor. Biomaterials 53, 621–633. doi:10.1016/j.biomaterials.2015.02.121 25890758 PMC4405661

[R89] Spitzer MH, and Nolan GP (2016). Mass cytometry: single cells, many features. Cell. 165 (4), 780–791. doi:10.1016/j.cell.2016.04.019 27153492 PMC4860251

[R90] Ståhl PL, Salmén F, Vickovic S, Lundmark A, Navarro JF, Magnusson J, (2016). Visualization and analysis of gene expression in tissue sections by spatial transcriptomics. Science 353 (6294), 78–82. doi:10.1126/science.aaf2403 27365449

[R91] Stephenson M, and Grayson W (2018). Recent advances in bioreactors for cell-based therapies. F1000Research 7, 517. doi:10.12688/f1000research.12533.1 PMC5931275 29770207

[R92] Sussman JH, Kim N, Kemp SB, Traum D, Katsuda T, Kahn BM, (2024). Multiplexed imaging mass cytometry analysis characterizes the vascular niche in pancreatic cancer. Cancer Res. 84 (14), 2364–2376. doi:10.1158/0008-5472.CAN-23-2352 38695869 PMC11250934

[R93] Syverud BC, Nagrath S, and Larkin LM (2018). Label-free, high-throughput purification of satellite cells using microfluidic inertial separation. Tissue Eng. Part C. Methods 24 (1), 32–41. doi:10.1089/ten.tec.2017.0316 28946802 PMC5756937

[R94] Tang F, Barbacioru C, Wang Y, Nordman E, Lee C, Xu N, (2009). mRNA-Seq whole-transcriptome analysis of a single cell. Nat. Methods 6 (5), 377–382. doi:10.1038/nmeth.1315 19349980

[R95] Thurner M, Asim F, Garczarczyk-Asim D, Janke K, Deutsch M, Margreiter E, (2018). Development of an *in vitro* potency assay for human skeletal muscle derived cells. PLOS ONE 13 (3), e0194561. doi:10.1371/journal.pone.0194561 29566057 PMC5864011

[R96] Tzimorotas D, Solberg NT, Andreassen RC, Moutsatsou P, Bodiou V, Pedersen ME, (2023). Expansion of bovine skeletal muscle stem cells from spinner flasks to benchtop stirred-tank bioreactors for up to 38 days. Front. Nutr 10. doi:10.3389/fnut.2023.1192365 PMC10442166 37609488

[R97] van der Schaft DWJ, Seftor REB, Seftor EA, Hess AR, Gruman LM, Kirschmann DA, (2004). Effects of angiogenesis inhibitors on vascular network formation by human endothelial and melanoma cells. JNCI J. Natl. Cancer Inst 96 (19), 1473–1477. doi:10.1093/jnci/djh267 15467037

[R98] Wang E, Feng B, Chen S, Su Z, and Chakrabarti S (2024). Differential microvascular endothelial cell responses in the retina in diabetes compared to the heart and kidneys, a spatial transcriptomic analysis. PLOS ONE 19 (12), e0310949. doi:10.1371/journal.pone.0310949 39739865 PMC11687817

[R99] Wang J, Broer T, Chavez T, Zhou CJ, Tran S, Xiang Y, (2022). Myoblast deactivation within engineered human skeletal muscle creates a transcriptionally heterogeneous population of quiescent satellite-like cells. Biomaterials 284, 121508. doi:10.1016/j.biomaterials.2022.121508 35421801 PMC9289780

[R100] Wei Z, Lei M, Wang Y, Xie Y, Xie X, Lan D, (2023). Hydrogels with tunable mechanical plasticity regulate endothelial cell outgrowth in vasculogenesis and angiogenesis. Nat. Commun 14 (1), 8307. doi:10.1038/s41467-023-43768-0 38097553 PMC10721650

[R101] Wolfe JT, Chen V, Chen Y, and Tefft BJ (2024). Identification of a subpopulation of highly adherent endothelial cells for seeding synthetic vascular grafts. J. Thorac. Cardiovasc. Surg 170, e27–e43. [Preprint]. doi:10.1016/j.jtcvs.2024.06.028 38972570 PMC11700231

[R102] Wood JA, Shah NM, McKee CT, Hughbanks ML, Liliensiek SJ, Russell P, (2011). The role of substratum compliance of hydrogels on vascular endothelial cell behavior. Biomaterials 32 (22), 5056–5064. doi:10.1016/j.biomaterials.2011.03.054 21501863 PMC3285275

[R103] Wu X, Peng Y, Li J, Zhang P, Liu Z, Lu H, (2022). Single-cell sequencing of immune cell heterogeneity in IgG4-Related disease. Front. Immunol 13. doi:10.3389/fimmu.2022.904288 PMC9184520 35693817

[R104] Young CS, Hicks MR, Ermolova NV, Nakano H, Jan M, Younesi S, (2016). A Single CRISPR-Cas9 Deletion Strategy that Targets the Majority of DMD Patients Restores Dystrophin Function in hiPSC-Derived Muscle Cells. Cell Stem Cell. 18 (4), 533–40. doi:10.1016/j.stem.2016.01.021 26877224 PMC4826286

[R105] Zhu H, Wu Z, Ding X, Post MJ, Guo R, Wang J, (2022). Production of cultured meat from pig muscle stem cells. Biomaterials 287, 121650. doi:10.1016/j.biomaterials.2022.121650 35872554

[R106] Zudaire E, Gambardella L, Kurcz C, and Vermeren S (2011). A computational tool for quantitative analysis of vascular networks. PLOS ONE 6 (11), e27385. doi:10.1371/journal.pone.0027385 22110636 PMC3217985

