# Supplementary material for: Cell manufacturing for cell-based tissue engineering: a focus on vascularized, skeletal muscle regeneration
Source: Front Chem Eng. Author manuscript; Available in PMC 2026 Mar 24. (PMC13008308; doi:10.3389/fceng.2025.1637075)
Supplement: Supplementary material [file NIHMS2154836-supplement-Supplementary_material.docx]

**Supplementary Table 1.** List of approved cellular and gene therapy products from the Office of Therapeutic Products at the U.S. Food and Drug Administration (Current as of May 25, 2025).

| **Name** | **Manufacturer** | **Type** | **Usage** |
| --- | --- | --- | --- |
| SYMVESS | Humacyte Global | Acellular Scaffold | Arterial Injury |
| LANTIDRA | CellTrans | Donor Cells | Type 1 Diabetes |
| OMISIRGE | Gamida Cell |  | Hematologic Malignancies |
| RYONCIL | Mesoblast |  | Graft vs. Host Disease |
| ALLOCORD | SSM Cardinal Glennon Children's Medical Center | Hematopoietic Progenitor Cells | Hematopietic Disease |
| Clevecord | Cleveland Cord Blood Center |  |  |
| Ducord | Duke University School of Medicine |  |  |
| Hemacord | New York Blood Center |  |  |
| HPC Cord Blood | Clinimmune Labs |  |  |
| HPC Cord Blood | MD Anderson Cord Blood Bank |  |  |
| HPC Cord Blood | LifeSouth Community Blood Centers |  |  |
| HPC Cord Blood | Bloodworks |  |  |
| REGENECYTE | StemCyte |  |  |
| LAVIV | Fibrocell Technologies | Patient Cells | Nasolabial Fold Wrinkles |
| PROVENGE | Dendreon |  | Prostate Cancer |
| ADSTILADRIN | Ferring Pharmaceuticals A/S | Viral Gene Therapy | Bladder Cancer |
| BEQVEZ | Pfizer |  | Hemophilia B |
| ELEVIDYS | Sarepta Therapeutics |  | Duchenne muscular dystrophy |
| HEMGENIX | CSL Behring |  | Hemophilia B |
| KEBILIDI | PTC Therapeutics |  | L Amino Acid Decarboxylase Deficiency |
| LUXTURNA | Spark Therapeutics |  | Retinal Dystrophy |
| ROCTAVIAN | BioMarin Pharmaceutical |  | Hemophilia A |
| VYJUVEK | Krystal Biotech |  | Dystrophic Epidermolysis Bullosa |
| ZOLGENSMA | Novartis Gene Therapies |  | Spinal Muscular Atrophy |
| CASGEVY | Vertex Pharmaceuticals | Hematopoietic Stem Cells/CRSPR | Sickle Cell Disease |
| LENMELDY | Orchard Therapeutics | Modified Patient Cells | Metachromatic Leukodystrophy |
| LYFGENIA | Bluebird bio |  | Sickle Cell Disease |
| SKYSONA | Bluebird bio |  | Cerebral Adrenoleukodystrophy |
| ZYNTEGLO | Bluebird bio |  | ß-Thalassemia |
| IMLYGIC | BioVex | Modified Virus | Melanoma |
| AMTAGVI | Iovance Biotherapeutics | T Cell Immunotherapy | Melanoma |
| ABECMA | Celgene Corporation | T Cell Immunotherapy/CART | Multiple Myeloma |
| AUCATZYL | Autolus |  | Acute Lymphoblastic Leukemia |
| CARVYKTI | Janssen Biotech |  | Multiple Myeloma |
| KYMRIAH | Novartis Pharmaceuticals |  | Acute Lymphoblastic Leukemia |
| TECARTUS | Kite Pharmaceuticals |  | Mantle Cell Lymphoma/Acute Lymphoblastic Leukemia |
| YESCARTA | Kite Pharma |  | Large B-cell Lymphoma/Follicular Lymphoma |
| BREYANZI | Juno Therapeutics | T Cell Immunotherapy/CART + CD19 Surface Mod | Large B-cell lymphoma |
| TECELRA | Adaptimmune | T Cell Immunotherapy/CART + TCR Surface Mod | Synovial Sarcoma |
| GINTUIT | Organogenesis | Encapsulated Cells | Mucogingival Conditions |
| MACI | Vericel |  | Cartilage Defects |
| RETHYMIC | Enzyvant Therapeutics |  | Congenital Athymia |
| STRATAGRAFT | Stratatech |  | Skin Burns |
| ENCELTO | Neurotech Pharmaceuticals | Encapsulated Modified Cells | Idiopathic Macular Telangiectasia Type 2 |
